# Phosphatidylcholine Cation—Tyrosine π Complexes: Motifs for Membrane Binding by a Bacterial Phospholipase C

**DOI:** 10.3390/molecules27196184

**Published:** 2022-09-21

**Authors:** Mary F. Roberts, Anne Gershenson, Nathalie Reuter

**Affiliations:** 1 Department of Chemistry, Boston College, Chestnut Hill, MA 02467, USA; 2 Department of Biochemistry and Molecular Biology, University of Massachusetts, Amherst, MA 01003, USA; 3 Computational Biology Unit, Department of Informatics and Chemistry, University of Bergen, 5020 Bergen, Norway

**Keywords:** phosphatidylinositol, PI-specific phospholipase C, phosphatidylcholine, NMR relaxometry, molecular dynamic simulations, cation-π interaction, allosteric effector

## Abstract

Phosphatidylinositol-specific phospholipase C (PI-PLC) enzymes are a virulence factor in many Gram-positive organisms. The specific activity of the *Bacillus thuringiensis* PI-PLC is significantly increased by adding phosphatidylcholine (PC) to vesicles composed of the substrate phosphatidylinositol, in part because the inclusion of PC reduces the apparent K_d_ for the vesicle binding by as much as 1000-fold when comparing PC-rich vesicles to PI vesicles. This review summarizes (i) the experimental work that localized a site on *Bt*PI-PLC where PC is bound as a PC choline cation—Tyr-π complex and (ii) the computational work (including all-atom molecular dynamics simulations) that refined the original complex and found a second persistent PC cation—Tyr-π complex. Both complexes are critical for vesicle binding. These results have led to a model for PC functioning as an allosteric effector of the enzyme by altering the protein dynamics and stabilizing an ‘open’ active site conformation.

## 1. Introduction

**Figure 1:**
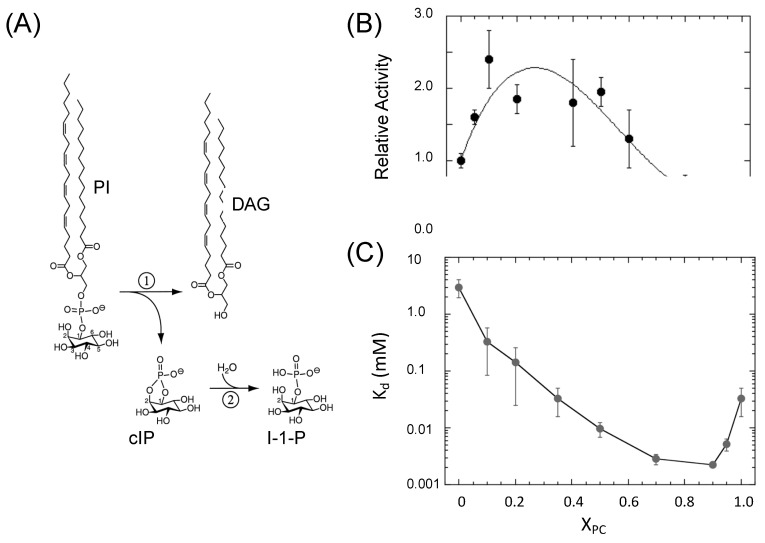
*Bt*PI-PLC chemical reaction and the effect of POPC on enzymatic activity and apparent dissociation constants. (**A**) Reaction catalyzed by PI-PLC produces DAG and cIP, the latter being a stable intermediate eventually hydrolyzed to I-1-P. (**B**) Enzyme activity towards PI in SUVs with POPC is shown as a function of mole fraction PC, X_PC_, under conditions where >75% of the enzyme is bound to vesicles. (**C**) Binding of PI-PLC to DOPG/POPC bilayers as a function of X_PC_. Graphs (**B**,**C**) are adapted with permission from Biochemistry 2009, 48, 6835–6845. Copyright (2009) American Chemical Society.

Bacterial phosphatidylinositol-specific phospholipase C (PI-PLC) enzymes are virulence factors secreted by Gram-positive organisms. For *Bacillus* sp., their role is to downregulate the host immune response by releasing glycosylphosphatidylinositol (GPI) anchored proteins from the cell surface, generating diacylglycerol (DAG) along with the soluble glycosylated protein [1,2]. In terms of the structure and mechanisms, most of the studies of PI-PLC enzymes from *Bacillus* sp. have used phosphatidylinositol (PI) as the substrate, rather than a GPI-linked protein. A notable exception is the work by Lehto and Sharom on the kinetics of cleavage of a purified GPI-anchored protein that exhibits much of the kinetic behavior observed with PI as the substrate, confirming the use of PI as a substitute for GPI-anchored proteins [3,4,5]. The PI-PLC catalyzed reaction for the hydrolysis of PI to inositol-1-phosphate, shown in Figure 1A, occurs via a general acid–general base mechanism where the PI is first cleaved to membrane-soluble diacylglycerol (DAG) and *myo*-inositol 1,2-(cyclic)phosphate (cIP), the latter being too polar to partition into membranes. The cIP can also be hydrolyzed by the enzyme to *myo*-inositol-1-phosphate. However, cIP is a poor substrate with a low k_cat_ (12 s^−1^) and a very high K_m_ (90 mM) [6]. The relatively small size of PI-PLC (34.8 kDa) and the ability to separate its activity toward interfacial (PI) and soluble (cIP) substrates make it a good system to study the detailed mechanism of the protein binding to different membranes and to explore how specific membrane components alter enzymatic activity. 

Early work showed that PI presented in small unilamellar vesicles (SUVs) or solubilized in Triton X-100 micelles was a poor substrate for the *B. thuringiensis* PI-PLC (*Bt*PI-PLC). With PI SUVs, k_cat_ and K_m_ were 73 s^−1^ and 2.6 mM, respectively [7]. However, the *Bt*PI-PLC phosphotransferase activity was significantly increased by including phosphatidylcholine (PC) in the vesicle or micelle along with PI [6,7]. Sphingomyelin, but not other phospholipids, also activated the enzyme, indicating that the phosphocholine group was critical for the activation of the enzyme. However, soluble PC molecules such as dibutyroyl-PC (diC_4_PC) were not activators, indicating that PC activation requires an interface. With PC/PI SUVs, the magnitude of the increased enzymatic activity depended on the mole fraction of the PC (X_PC_) as well as the total amount of phospholipids present. The data shown in Figure 1B used 10 mM PI SUVs, and additional POPC was added to increase the X_PC_. For that concentration of pure PI, the enzyme would be ~75% saturated. The inclusion of PC in the vesicles increased the specific activity more than two-fold at its maximum. The apparent K_d_, measured by fluorescence correlation spectroscopy [8], was comparable to the K_m_, indicating that for PI SUVs, the rate-limiting step was binding to the SUVs. As shown in Figure 1C, the apparent K_d_ for *Bt*PI-PLC binding to vesicles composed of dioleoylphosphatidylglycerol (DOPG) as the PI surrogate and 1-palmitoyl-2-oleoyl-PC (POPC) as the activator decreased ~1000-fold, with the tightest binding occurring in the PC-rich vesicles. The residence time of *Bt*PI-PLC on a PC-rich tethered SUV vesicle, measured with single-molecule fluorescence microscopy, is 380 ± 50 ms [9], allowing many rounds of PI cleavage on the vesicle surface.

The second step of PI cleavage, cIP hydrolysis, was also increased with the addition of PC SUVs [7] or short-chain PC micelles [6]. Again, the addition of other phospholipids (PS, PA, and PG) in the micelles or vesicles did not activate the enzyme. The cIP is either in solution or bound to the protein and not partitioned into the membrane. Therefore, interfacial PC is an allosteric effector of *Bt*PI-PLC. However, these kinetic and binding results do not differentiate between a distinct PC binding site on *Bt*PI-PLC or the nonspecific membrane perturbation effects that alter the conformation or dynamics of PI-PLC. While these results provided data on the importance of PC or sphingomyelin for *Bt*PI-PLC vesicle binding and activity, they did not elucidate the molecular mechanism for the enhanced binding and activity. 

## 2. Experimental Results—Characterization of Specific PC Binding Site(s) on *Bt*PI-PLC

### 2.1. B. thuringiensis PI-PLC, a Member of the TIM Barrel Superfamily

**Figure 2:**
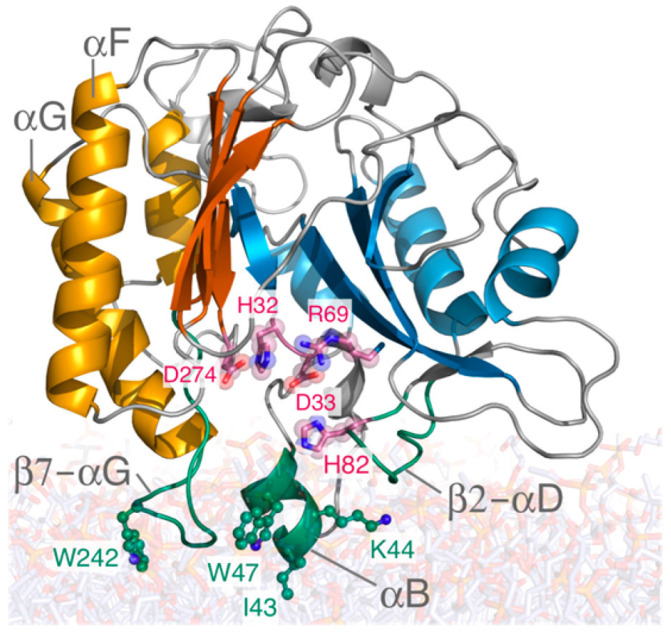
Snapshot of the structure and lipid binding of *B. thuringiensis* PI-PLC from all-atom molecular dynamics (MD) simulations [13]. The X-box and variable region of the βα barrel are in shades of blue and orange, respectively. The active site is shown as pink sticks for key catalytic residues (His32, Asp33, Arg69, His82, and Asp274); the IBS is green and includes hydrophobic Ile43, Trp47, Trp242, and cationic Lys44. Reprinted with permission from Roberts et al. (2018) Chem. Rev. 2018, 118, 8435–8473. Copyright (2018) American Chemical Society.

The crystal structures of *Bacillus* sp. PI-PLCs, the recombinant proteins, and the various mutants (see Table 1 in reference [10]) all show a distorted β-barrel structure. While a crystal structure for wild-type *Bt*PI-PLC is not available, the *B. cereus* enzyme only differs by a few amino acids, and its structure [11,12] can be used to model *Bt*PI-PLC. The structure of the enzyme, shown in Figure 2 with the key regions identified by mutating and assessing the loss of activity or membrane binding, is from molecular dynamics simulations of the enzyme docked on a dimyristoyl-PC (DMPC) bilayer [13]. The active site residues are in red. Trp47, in helix B, and Trp242, in the β7–αG rim loop, insert into the membranes and are key components of the interfacial binding site (IBS) of the protein. 

### 2.2. Experimental Evidence for a Specific PC Binding Site on B. thuringiensis PI-PLC 

While *Bt*PI-PLC’s requirement for PC (or sphingomyelin) in an interface for optimal cleavage of PI (or optimal hydrolysis of cIP) is suggestive of a specific binding site for that zwitterionic phospholipid, identifying such a site is difficult. For many other amphitropic proteins that bind to membranes via specific phospholipid headgroups (e.g., PH domains that bind different phosphoinositides), a soluble polar group alone can often bind to the protein well enough to pinpoint the lipid binding site. However, soluble phosphocholine, glycerophosphocholine, and diC_4_PC have very a low affinity for *Bt*PI-PLC [6] (also see the Appendix for NMR data that show the very poor binding of diC_4_PC to spin-labeled D205C). As an interface is needed for the PC activation of *Bt*PI-PLC, other approaches were necessary. 

The crosslinking agent 1-ethyl-3-[3-dimethylaminopropyl]carbodiimide hydrochloride (EDC) forms an amide bond between the nearby side chains of lysine and acidic residues (Asp, Glu). The somewhat naïve thought behind the experiment was that crosslinking the protein in the presence of an interface might trap it in an active form. *Bt*PI-PLC was mixed with diheptanoyl-PC (diC_7_PC) micelles rather than a bilayer PC interface since the crosslinked micelle/protein complex could be extensively dialyzed at pH 7 to remove all but the most tightly bound diC_7_PC molecules. Crosslinked and dialyzed *Bt*PI-PLC exhibited a mass increase of ~1 kDa (exptl. error ± 0.15 kDa), equivalent to two tightly bound diC_7_PC molecules [14]. If the dialysis was performed at pH > 8, the mass increase was not detected, indicating that crosslinking stabilized a conformation of the protein where the affinity for the two diC_7_PC molecules was very high around neutral pH, but not in basic conditions. In the absence of EDC, there was no excess mass observed after mixing the *Bt*PI-PLC with (diC_7_PC) and then dialyzing it. The crosslinked protein with the two lipid molecules bound was more than twice as active towards PI/diC_7_PC as the uncrosslinked protein. Two surface tryptophan residues, Trp47 in helix B and Trp242 in the ←β7–αG loop, are important for vesicle binding [15,16]. If either one is removed, the crosslinked and then dialyzed protein has a mass for only a single tightly bound diC_7_PC molecule. 

With unaltered recombinant *Bt*PI-PLC, one sees a substantial broadening of the diC_7_PC ^31^P resonance when the enzyme is added [17]. This is due to the exchange of PC molecules between the monomers in solution, the micelles, and the potential *Bt*PI-PLC binding sites, as well as the increases in diC_7_PC micelle size upon *Bt*PI-PC binding. Mixing the dialyzed crosslinked *Bt*PI-PLC with diC_7_PC micelles did not lead to line broadening, a result implying that the tightly bound diC_7_PC molecules are not in fast exchange with the added diC_7_PC micelles or monomers. These crosslinking experiments indicated that two PC molecules can be tightly associated with the *Bt*PI-PLC but provided no information on where they were bound. There are nine Lys–Asp/Glu pairs within crosslinking distance, making identification of where the PC molecules were bound problematic. 

The interaction of the protein with the phospholipid vesicles required a different approach to identify a PC binding site. While many experimental methods have been used to characterize the binding of proteins to membranes, most do not directly identify binding sites (see [10] for an extensive review of these methods). High resolution ^31^P relaxometry (also referred to as shuttle field-cycling ^31^P NMR relaxometry) is a very useful but not well known technique that can be used to identify and characterize specific protein interactions with phospholipids in small unilamellar vesicles or micelles. This type of relaxometry measures the spin-lattice/longitudinal relaxation rate (R_1_ = 1/T_1_) over a wide range of magnetic fields by rapidly shuttling the sample, excited at a high field, to different positions in the bore of the superconducting magnet for relaxation at a lower field (B_relax_). The range of B_relax_ used for studying *Bt*PI-PLC binding to small vesicles was 11.7 T down to 0.003 T [18,19]. After a time at B_relax_ that is varied, the sample is returned to the probe for a signal readout. Phospholipids in bilayers have many different motions covering a wide range of timescales [20] that alter the orientation or interactions of the phospholipid ^31^P–^1^H dipoles. Different motions will give rise to nuclear magnetic relaxation dispersions (NMRDs) with correlation times related to the specific motion. The field dependence of ^31^P R_1_ is the sum of all the individual NMRDs. 

**Figure 3:**
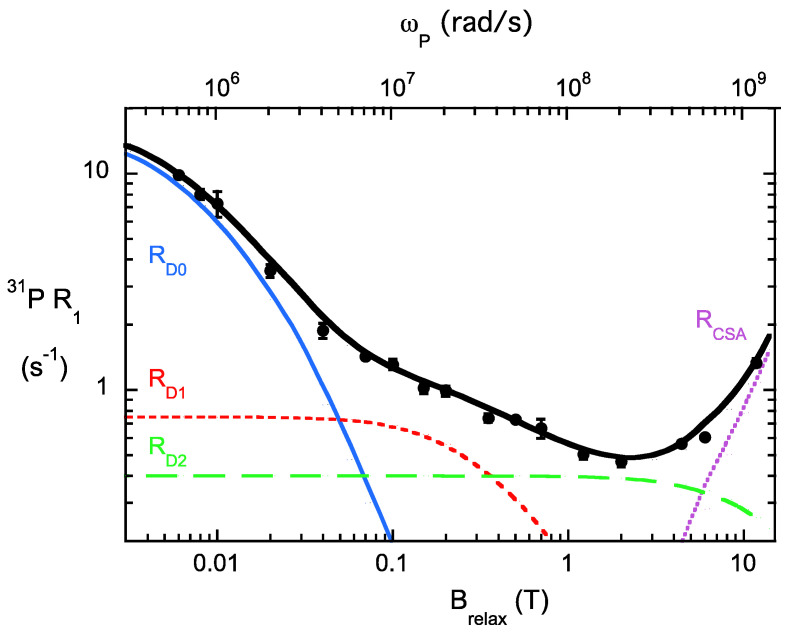
Dependence of R_1_ for the POPC ^31^P resonance in SUVs mixed 1:1 with d_3_-DOPMe with relaxation magnetic field, B_relax_. The deconvolution of the data into three dipolar NMRDs and a single high-field NMRD due to the ^31^P chemical shift anisotropy is shown. The Larmor angular frequency, ω_P_, corresponding to γ_P_ B_relax_, is shown on the top x-axis. Reprinted with permission from Roberts et al. (2021) J. Phys. Chem. B, 125, 8827–2238, Copyright 2021, American Chemical Society.

Figure 3 shows the ^31^P field cycling profile for POPC in POPC/d_3_-DOPMe (1:1) SUVs as a function of B_relax_. The dependence of R_1_ on the Larmor angular frequency ω_P_ (where ω_P_ = γ_P_ B_relax_) is also shown. The variation of the ^31^P R_1_ with B_relax_ is characterized by three dipolar NMRDs, labeled R_D0_, R_D1_, and R_D2_. R_D0_, occurring at the lowest fields, has a correlation time, τ_D0_, that reflects the overall tumbling of the aggregate (the bigger the particle, the longer the τ_D__0_) conflated with the translational movement of the phospholipids. For SUVs, τ_D0_ is typically 0.5–1.5 µs. For R_D1_, τ_D1_ is in the 10–15 ns range; this NMRD arises from the axial/wobble motions of the phospholipid molecules. R_D2_ is the result of fast dipolar motions that are very localized, e.g., changes in dihedral angles. These occur on sub-ns timescales. R_D2_ is partially obscured by a fourth NMRD, R_CSA_, which is from the fast motions associated with the chemical shift anisotropy of the ^31^P. The measurements of R_1_ at the high fields of modern spectrometers primarily reflect the fast motions. The partitioning of a protein onto the bilayer can alter some or all of these ^31^P motions. For example, the differential changes in R_D0_ and τ_D0_ for POPC/DOPG SUVs when *Bt*PI-PLC is added provide information on the translational diffusion of each phospholipid in the plane of the membrane [21]. 

The average distance of a phospholipid ^31^P from the specific regions of the transiently bound protein is provided by introducing a cysteine at a specific site on the protein and spin-labeling it. This provides an unpaired electron that is a much more potent relaxer than the -OCH_n_- protons linked to ^31^P that normally dominate the dipolar relaxation of ^13^P. In these experiments, the ratio of each phospholipid in the outer monolayer to *Bt*PI-PLC was typically between 230 and 260 (the range is because the SUVs are not a single size and but cover a range presenting different ratios of phospholipids in the outer surface [8]). At this ratio, there are few proteins on a given small vesicle; so, protein/protein interactions (a potential complication if both are spin-labeled) are unlikely to occur. Enhanced relaxation of the phospholipid ^31^P caused by a nearby spin label on the protein implies that a phospholipid must occupy that site for the correlation time of that NMRD. Therefore, we use changes in R_D0_, which has the longest correlation time, to define a specific phospholipid/protein complex. 

*Bt*PI-PLC is an ideal candidate for this technique since it lacks cysteine residues. A cysteine can be introduced at many different sites on the protein, and as long as the enzymatic activity is not altered by the presence of the spin-labeled Cys, the increased ^31^P R_1_ can provide an averaged distance of the ^31^P to the electron (r_P__-e_). This is obtained by subtracting the R_1_ profile for the vesicles with the same amount of unlabeled protein from the profile of ^31^P R_1_ for the vesicles with spin-labeled protein as a function of B_relax_. The resultant ΔR_D0_ NMRD provides the correlation time for the ^31^P–electron interaction, τ_P__-e_, and the maximum relaxation rate, R_P-e_(0). As the ^31^P–^1^H contribution to the relaxation has been subtracted (Equation (1)), the ΔR_D0_ NMRD can be fitted with only the ^31^P spectral density function (Equation (1)) and a constant, c, equal to R_D1(P-e)_(0)+R_D2(P-e)_(0). The ratio τ_P__-e_/ΔR_P-e_(0) and a correction for how much of the ligand is bound to the protein (estimated from the K_d_) are used to obtain the distance of a given spin label to the phosphorus atom of each phospholipid (Equation (2)).

(1) $$\Delta R_{P-e}=R_{P-e}\left(0\right)/\left(1+{\omega_{P}}^{2}{\tau_{P-e}}^{2}\right)+c$$

(2) $${r_{P-e}}^{6}=(\left[\mathrm{protein}*\mathrm{ligand}\right]/\left[\left(2/3\right)\mathrm{total}\;\mathrm{ligand}\right]\;\left(\frac{\tau_{P-e}}{\Delta R_{P-e}\left(0\right)}\right){\left(\frac{\mu }{4\pi }\right)}^{2}{\left(\frac{h}{2\pi }\right)}^{2}\;{\gamma_{P}}^{2}\gamma_{e}$$

Note that the 2/3 in Equation (2) accounts for the fact that the protein binds to the outer leaflet of the vesicle, which for our SUVs contains approximately two-thirds of the total phospholipids. A series of r_P__-e_ for *Bt*PI-PLC spin-labeled at different sites provides constraints for the ^31^P–electron interaction that, together with computer modeling, can localize specific phospholipid binding sites [22]. More details on the method are found in the Section 6. 

**Figure 4:**
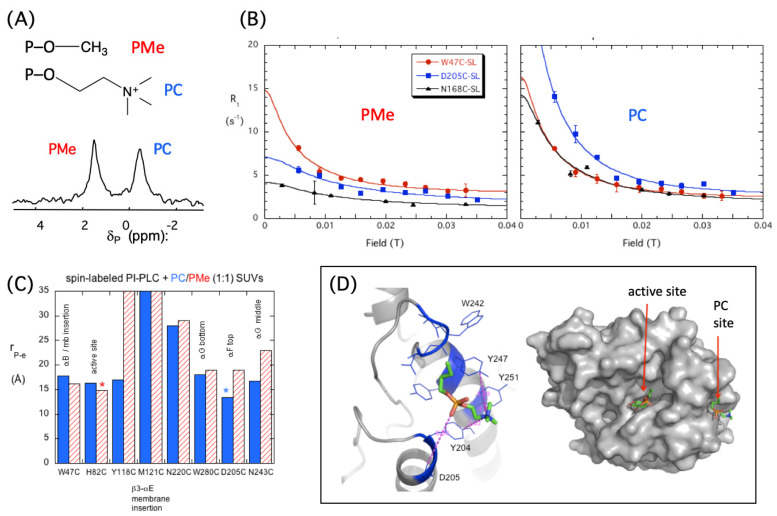
^31^P shuttle field cycling relaxometry of POPC/DOPMe SUVs interacting with spin-labeled BtPI-PLC. (**A**) Polar head groups of each phospholipid and the ^31^P spectrum at 11.7 T. (**B**) The R_D0_ NMRD for each phospholipid with three different spin-labeled (SL) BtPI-PLC: W47C-SL, D205C-SL, and N168C-SL. (**C**) The r_P-e_ extracted from the field cycling increase in R_1_ for POPC (blue) and DOPMe (red) with the structural region of each spin label indicated. (**D**) The likely location of the POPC most affected by D205C is one of the Tyr in the N-terminal portion of helix G. The overall spatial relationship of this site to the active site is also indicated. Figure 4B is modified, and Figure 4D is reprinted from Pu et al. J. Biol. Chem. 2010, 285, 26916–26920. Copyright 2015, American Society for Biochemistry and Molecular Biology.

Figure 4A provides a ^31^P spectrum for the dioleoylphosphatidylmethanol (DOPMe) and POPC in the same SUVs as well as a polar headgroup structure. Figure 4B illustrates the effect of the three spin labels picked to cover the different regions on the protein. Anionic DOPMe, the substrate surrogate, is a good inhibitor that is not hydrolyzed by the enzyme over the 24 h of the field cycling experiment. The DOPMe and POPC ^31^P resonances exhibit different R_D0_ profiles, indicating different binding sites and proximities to the spin label (Figure 4B). Subtracting the control (vesicles mixed with protein lacking a spin label) and analyzing the R_D0_ region with Equation (1) provides τ_P-e_ and ΔR_P-e_(0). The ratio τ_P-e_/ΔR_P-e_(0) is related to r_P-e_^6^ (Equation (2)). Figure 4C shows the r_P-e_ for POPC and DOPMe that has been extracted for the different spin-label positions in *Bt*PI-PLC. Each Cys mutation is annotated as to the structural feature it is in or near.

For DOPMe, the strongest paramagnetic relaxation effect is with the spin label at H82C (near or in the active site). It is also relaxed by a spin label in helix B (W47C). The strongest relaxation effect for the PC is when the spin label is at the top of helix F (attached to D205C). This region of the protein is relatively far from the active site (Figure 4D). The nearby N-terminal end of helix G has an unusual composition with a string of four tyrosine residues, Tyr246, Tyr247, Tyr248, and Tyr251, whose mutation to Ser or Ala causes a large loss in binding affinity [13]. One or more of these could form a cation-π complex with the PC trimethylammonium moiety. Such a PC binding site in this region would be 15–17 Å from the active site. 

If a single PC stayed in a cation-π site for the entire residence time of the protein on the vesicle (380 ms), it would be in a slow exchange with the bulk POPC in the SUV, and it would not be detected. As the relaxation of the ^31^P by the spin label is observed, there is a fast exchange between the enzyme-bound and the bulk phospholipid environments for both POPC and DOPMe. Again, this means that while the protein is anchored on the vesicle, the PC molecules are moving back and forth from the bulk bilayer to the enzyme binding site.

**Figure 5:**
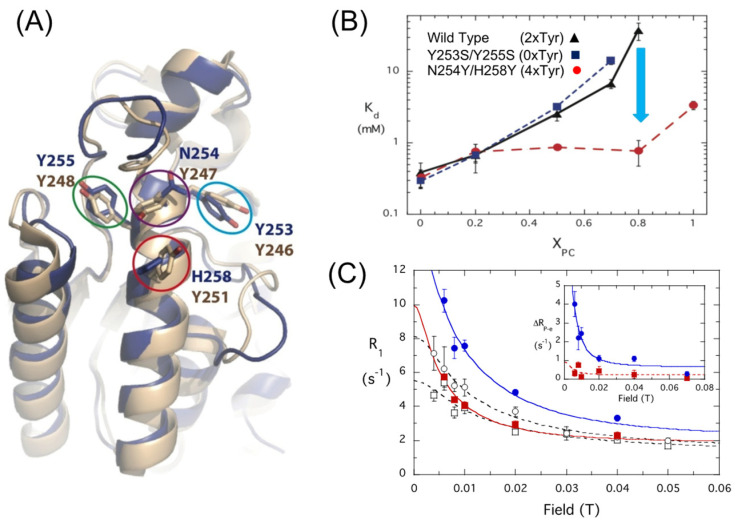
Structure of *S. aureus* PI-PLC compared to *B. thuringiensis* PI-PLC; the effects of adding two Tyr residues on the apparent K_d_ for PC-rich vesicles and ^31^P field cycling evidence for introduction of a PC cation–Tyr-π interaction in SaPI-PLC N254Y/H258Y. (**A**) Comparison of *Sa*PI-PLC structure (dark blue) and *Bt*PI-PLC structure (brown). The ovals compare the cluster of Tyr residues in *Bt*PI-PLC with the wild-type *Sa*PI-PLC. (**B**) Apparent K_d_ (at pH 6.5) for *Sa*PI-PLC wild type (triangle), Y253S/Y255S (square), and N254Y/H258Y (circle). The arrow emphasizes the 30-fold drop in K_d_ for N254Y/H258Y compared to wild type. (**C**) Effect of spin label attached to D213C *Sa*PI-PLC (0.5 mg/mL) on ^31^P R_1_ of POPC (5 mM)/DOPMe (5 mM) SUVs as a function of the relaxation field. Filled symbols (and the solid line fits) are for PC (blue circles) and DOPMe (red squares) with spin-labeled protein. Open symbols are for the control where the same SUVs were used but using D213C/N254Y/H258Y without a spin label. The inset shows ΔR_1_ due to the spin label. Parts (**B**,**C**) are adapted from He et al. J. Biol. Chem. 2015, 290, 19334–19342. Copyright 2015, American Society for Biochemistry and Molecular Biology.

Further experimental evidence that the site identified for the PC was indeed a PC cation–Tyr complex was provided by engineering a site to mimic that of *Bt*PI-PLC in the PI-PLC from *Staphylococcus aureus*. The enzyme from *S. aureus* (*Sa*PI-PLC) has a similar structure to the *Bt*PI-PLC (Figure 5A) but very poor affinity for PC-rich SUVs [23]. It lacks two of the four Tyr residues in helix G. Removing the remaining two Tyr (Y253S/Y255S) has little effect on the affinity of the enzyme for the PG/PC vesicles (Figure 5B). At X_PC_ = 0.7, there is only a 2-fold increase in K_d_. In contrast, adding the two ‘missing’ Tyr (N254Y/H258Y) decreased the K_d_ for *Sa*PI-PLC N254Y/H258Y dramatically, around 30-fold at X_PC_ = 0.8, and increased the PC ^31^P R_1_ at low fields for POPC but not DOPMe (Figure 5C). 

The final experimental evidence that vesicle binding was mediated by a PC cation–Tyr-π complex was the introduction of 3,5-difluorotyrosine (Y-F_2_) into specific Tyr sites in *Sa*PI-PLC [24]. If H258Y is forming a cation-π complex, then the K_d_ for N254Y/H258Y-F_2_ should increase substantially for PC-rich SUVs because the negative charge in the aromatic ring is reduced by the attached fluorine atoms. If instead that tyrosine is inserted into the membrane, then the introduction of the two fluorine atoms in the Tyr ring will make the side chain more hydrophobic, which will decrease K_d_. At X_PC_ = 0.8, the K_d_ for N254Y/H258Y-F_2_ is 10-fold higher than for N254Y/H258Y, which is consistent with a cation-π complex. The experiments with Y-F_2_ replacing specific Tyr in *Bt*PI-PLC indicate that one or more PC cation–Tyr-π complexes are critical to the binding of that enzyme on the membranes.

## 3. Computational Results—Identification of PC Binding Sites on *Bt*PI-PLC

### 3.1. Transient Opportunistic and Very Specific PC Cation–PI-PLC Tyr-π Interactions 

The experimental data clearly supported the formation of a cation-π complex between PC and *Bt*PI-PLC and localized a plausible PC binding site on the protein where one or more of a string of Tyr residues could form these complexes. For a more detailed view of the potential PC cation–Tyr-π sites, multiple 500 ns all-atom molecular dynamics simulations were run of the *Bt*PI-PLC binding to dimyristoylphosphatidylcholine/dimyristoylphosphatidylglycerol (DMPC/DMPG) and DMPC bilayers [13,25]. 

**Figure 6:**
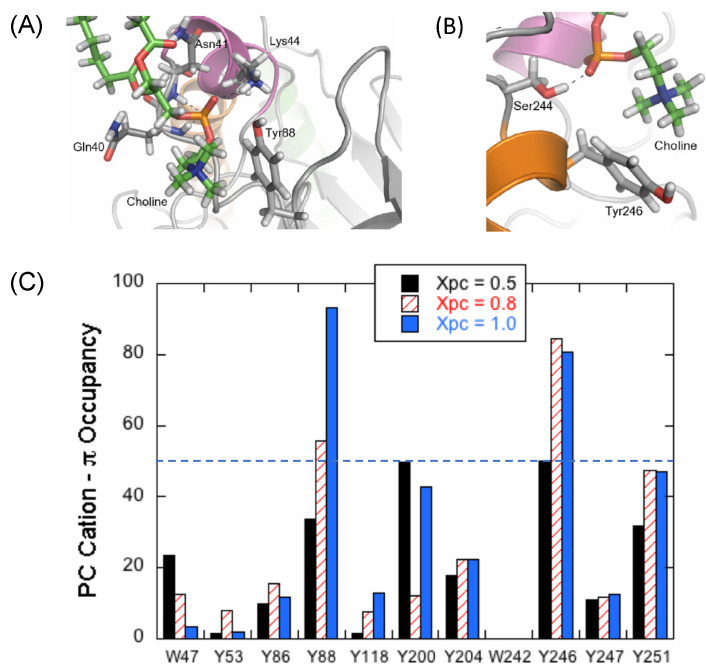
Occupancy of PC cation–Tyr-π complexes identified in MD simulations depends on X_PC_. Snapshots of cation-π interactions at X_PC_ = 1 for (**A**) Tyr88 and (**B**) Tyr246 (representative frames taken between 400 and 500 ns). (**C**) Occupancy of cation-π sites as a function of X_PC_. Both (**A**,**B**) are reprinted with permission from Grauffel et al., J. Am. Chem. Soc. 2013, 135, 5740–5750. Copyright 2013, American Chemical Society.

One of the surprising results from these initial simulations was that many transient DMPC cation–Tyr-π complexes were formed during the simulation. However, two cation-π complexes, with Tyr88 and Tyr246, existed for more than 80% of the simulation time with the same phospholipid. Tyr88 is near helix B, and Tyr246 is in the N-terminal region of helix G, both of which are features known to be important for binding to membranes. Snapshots of two of these complexes are shown in Figure 6A,B. They also persist in mixed DMPC/DMPG bilayers at X_PC_ = 0.8 and 0.5 (Figure 6C) [25]. The DMPC complex with Tyr246 is consistent with the r_P-e_ obtained experimentally by high-resolution field cycling [22]. The Tyr88 complex was unexpected, but its contribution to the POPC R_1_ does fit the shorter than expected r_P-e_ obtained for the POPC when the protein was spin-labeled on helix B or the active site residues (both regions fairly far from Tyr246 if that were the only cation-π complex). The multiplicity of the PC cation–Tyr-π complexes in the *Bt*PI-PLC simulations also suggests why the decrease in K_d_, for the engineered *Sa*PI-PLC N254Y/H258Y binding to X_PC_ = 0.8 SUVs was to only 0.7 mM rather than the 2 µM for *Bt*PI-PLC. There is no analogue of Tyr88 in the *S. aureus* enzyme.

Two other tyrosine residues, Tyr200 and Tyr251, also form transient cation-π complexes that are reasonably occupied. Tyr200 is in the *Bt*PI-PLC active site where it has the role of stabilizing the bound PI inositol ring [11,12]. The DMPC forming a choline cation/Tyr200-π complex is halfway out of the bilayer and into the active site. *Bt*PI-PLC enzymatic activity decreases at high X_PC_ [7], and the activator PC binding in the active site could be partially responsible for the activity decrease. It is worth noting that DMPG was not observed in the active site in any of the simulations. Kinetics studies with different phospholipid headgroups indicate that PG has a weaker affinity for *Bt*PI-PLC than other anionic phospholipids with smaller headgroups [7,17]. The glycerol moiety is fairly flexible, unlike the inositol ring, which presents a face of axial protons able to interact with Tyr200. Anionic phospholipid inhibitors with small headgroups, such as phosphatidylmethanol or phosphatidic acid, are unlikely to interact directly with Tyr200, but they could interact with cationic Arg69 or protonated His32 or His82 in the active site to effectively inhibit the enzyme. 

### 3.2. A Computational Approach to Estimating ΔG^0^ _bind_ Provides a Membrane Desorption Pathway for BtPI-PLC

**Figure 7:**
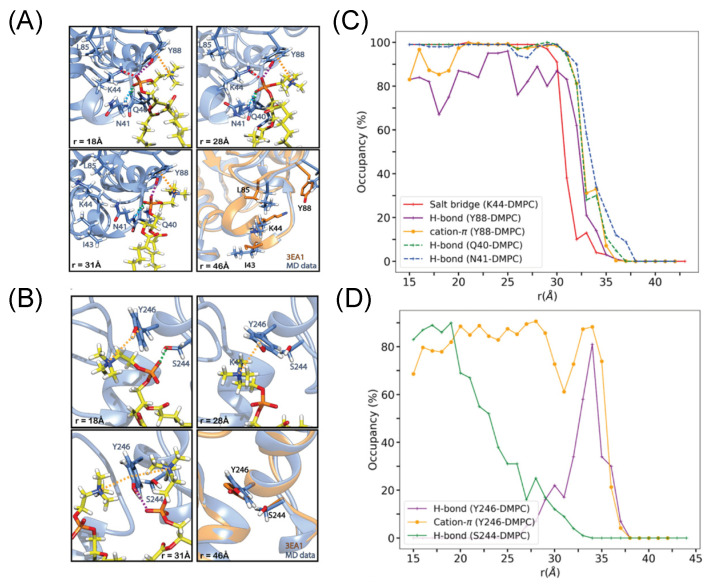
The Tyr88 and Tyr246 cation-π interactions with DMPC as *Bt*PI-PLC is separated from the membrane. The free energy minimum for bound *Bt*PI-PLC occurs at 18 Å. Snapshots of (**A**) Tyr88 and (**B**) TyrY246 interactions are shown at different distances from the membrane center during detachment from the DMPC bilayer. The *Bt*PI-PLC backbone, shown as blue ribbon, is aligned with a crystal structure backbone in orange. Selected side chains are shown as sticks colored by atom type; the DMPC, shown as sticks, has yellow C atoms. The dotted lines indicate the interactions between the protein and DMPC. The occupancies of the cation-π (orange), salt bridge (red), H-bonds (various colors indicated on each panel), for the (**C**) Y88 and (**D**) Tyr246 networks are plotted versus r_1_, the distance from the center of mass of the protein and that of the upper phosphate plane. Protein is fully desorbed by 38 Å. Reprinted with permission from Moutoussamy et al., (2022), J. Chem. Inf. Model (doi: 10.1021/acs.jcim.1c01543), Copyright 2022 the authors.

Recent work to calculate the absolute membrane binding free energy for *Bt*PI-PLC used a geometrical route and an atomistic force field to progressively detach the protein from the bilayer [26]. Along with a value for ΔG^0^ _bind_ (which agrees moderately well with the value from fluorescence correlation spectroscopy binding data for *Bt*PI-PLC binding to PC SUVs), the method provides atomic-level details that describe the membrane-bound protein and how interactions change during the desorption process. The two persistent cation-π complexes, involving Tyr88 and Tyr246, show distinct interactions with other nearby amino acids in the membrane-bound form, and these change as the protein is extracted (Figure 7). In the membrane-bound state, the stability of the PC-cation–*Bt*PI-PLC Tyr88-π complex is aided by a hydrogen bond between the Tyr–OH and the PC phosphate and three nearby residues interacting with the PC molecule: (1) Lys44 (in helix B) forms a salt bridge with the PC phosphate group, and (2) Gln40 and (3) Asn41 hydrogen bond with the DMPC (Figure 7A). 

**Figure 8:**
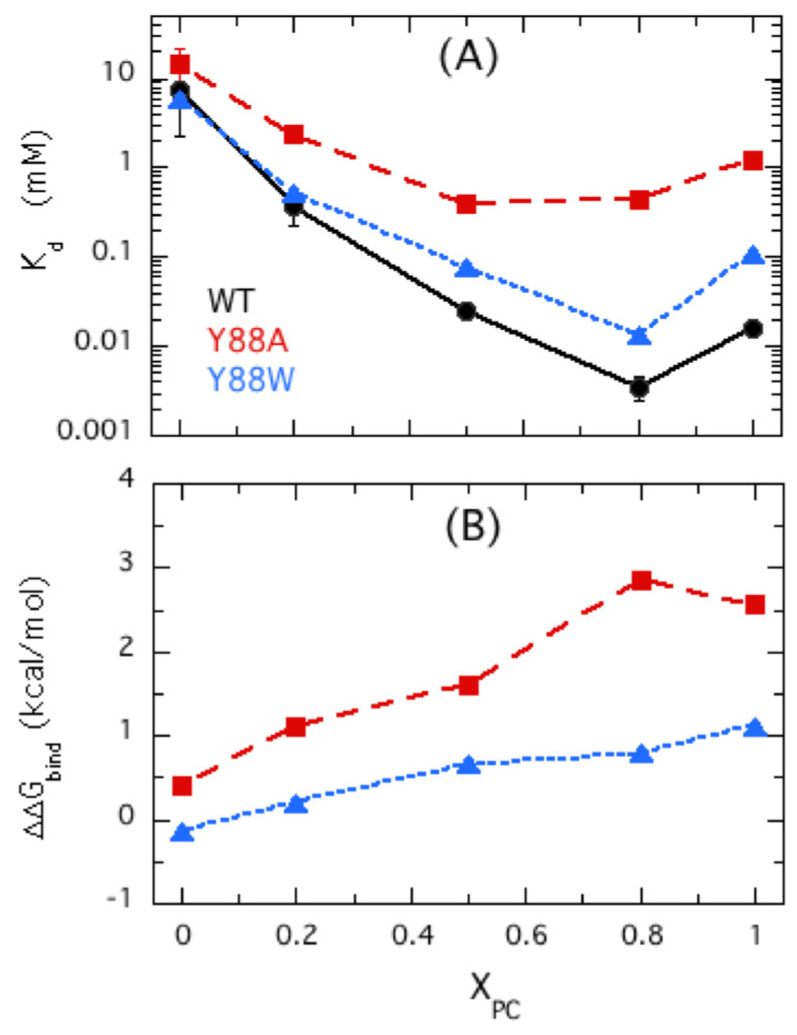
Effect of Tyr88 mutations on the K_d_ for binding to DOPMe/POPC SUVs and the change in ΔΔG^0^ caused by the mutation. (**A**) Apparent K_d_ values determined for unmutated BtPI-PLC (WT circles), Y88A (squares), and Y88W (triangles) as a function of X_PC_. (**B**) The change in binding free energy relative to wild-type BtPI-PLC caused by the mutation as a function of X_PC_.

There is limited experimental evidence consistent with this network of interactions stabilizing the Tyr88 cation-π complex. However, the variants Y88A and Y88W lend support for the formation of a hydrogen bond between the hydroxyl group of Tyr88 and the DMPC phosphate (Figure 8). The Y88A protein has lost one of the cation-π interactions and its K_d_ increases significantly with an increase in the ΔΔG^0^ _bind_ of +2.5 kcal/mol for binding pure DMPC bilayers (Figure 8). Y88W does recover some of the binding energy lost by Y88A, but the protein still does not bind as tightly (or more tightly, as expected for a choline cation–Trp-π complex) as unmodified *Bt*PI-PLC. The ΔΔG^0^ _bind_ for Y88W compared to wild-type PI-PLC is +1 kcal/mol, equivalent to the loss of a hydrogen bond. It is likely that a cation-π interaction is intact in Y88W, but the hydrogen bond between the aromatic indole ring and the DMPC phosphate is not present. The larger indole ring in this position and perhaps the misalignment of the N-H on the indole ring could preclude formation of the H-bond with the PC phosphate group. 

The network for the other persistent cation-π adduct is much less complex. The membrane-bound DMPC cation–Tyr246-π complex with a PC molecule is stabilized by a hydrogen bond between the Ser244 and the DMPC phosphate (Figure 7B). However, when the protein is pulled more than 5 Å away from the bilayer center, the occupancy of that hydrogen bond of Ser with the DMPC starts to decline (Figure 7D). At r = 27 Å, a new interaction appears. The tyrosine -OH group forms a hydrogen bond with the DMPC phosphate. This bidentate Tyr246 complex with DMPC stays intact until the protein is close to completely detaching. 

## 4. What Is the Allosteric Mechanism for PC Altering BtPI-PLC Enzymatic Activity?

It is clear that PC in a bilayer dramatically increases the affinity of *Bt*PI-PLC for surfaces by forming PC cation–tyrosine-π complexes that anchor the enzyme on the bilayer. These complexes would be the first to form when the protein makes initial contact with the bilayer and the last interactions to break as the protein is released from the bilayer. Lowering the apparent K_d_ makes an important contribution to improving enzyme specific activity, particularly for low concentrations of vesicles. As the assay conditions for Figure 1B had a near saturated enzyme, the two-fold increase in activity could reflect an increase in k_cat_. Rather than deal with complex models to sort out the interfacial kinetics, an easier approach is to use cIP, which has no affinity for phospholipid interfaces, as the substrate to test whether the PC/*Bt*PI-PLC complex does in fact alter k_cat_. For the cleavage of cIP in the absence of interfacial PC, the V_max_ and K_m_ for cIP are 20 µmol min^-1^ m^-1^ and 90 mM, respectively [6]. The presence of 8 mM diC_7_PC increases k_cat_ ~7-fold and decreases the K_m_ 3-fold. This corresponds to an increase in enzyme efficiency from 130 to 2600 s^−1^ M^−1^. Simulations found little change in the *Bt*PI-PLC structure in solution versus when bound to a bilayer [13]. A possible explanation for how PC, bound to the protein via two cation-π complexes, increases enzyme efficiency is that it alters the protein dynamics. 

The crystal structure of the *B. cereus* PI-PLC had weak intensity in the rim of the active site. Helix B, as well as the loop residues 237–243, was particularly weakly defined [11,12]. Principal component analysis of the MD simulations (20–50 ns) of *Bt*PI-PLC in aqueous solution [27] identified a clamshell-like motion with β-strands 1–5, along with associated loops and helices moving as one unit and strands 5b-8 moving as a second unit. This links the motions of helix B (residues 39–46) on one side of the clamshell with the loops of the N-terminal to helix F (residues 201–203) and helix G (residues 238–245). Both of these regions interact with the membranes, as assessed by mutagenesis and the MD simulations. The clamshell opens and closes over the active site and is observed in all simulations of the WT enzyme in solution. In the TIM barrel superfamily, a lid often controls access to the active site, and the loop between strand 7 and helix G is frequently associated with phosphate binding [28]. For *Bt*PI-PLC in the absence of PC, this type of motion of loops and helices could certainly limit the active site access. This could be a strong barrier for a water-soluble substrate such as cIP binding in the active site.

**Figure 9:**
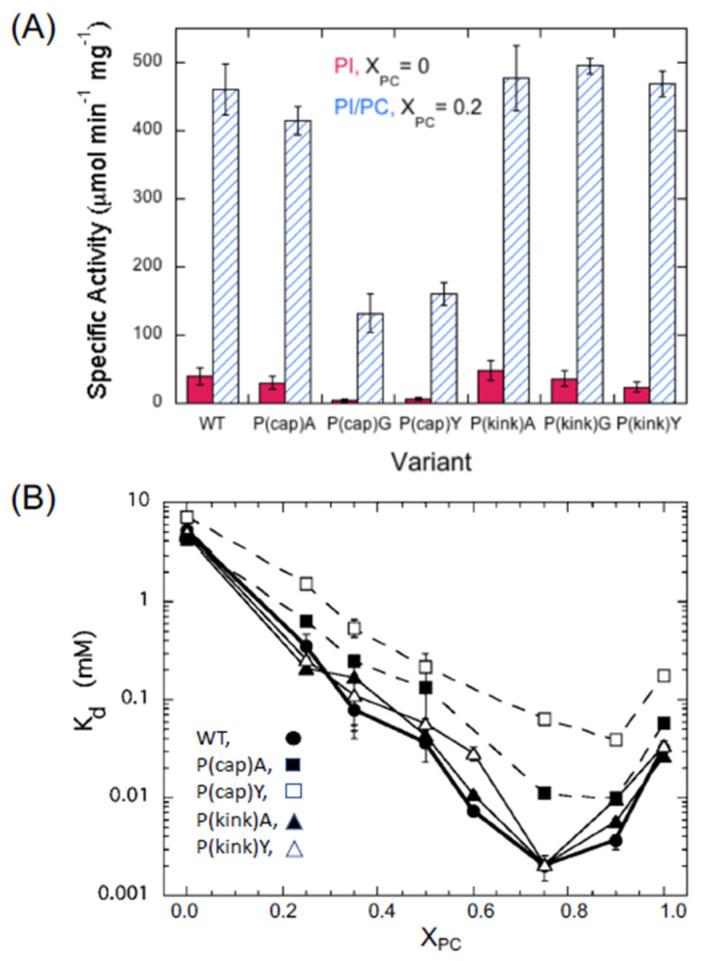
*Bt*PI-PLC P(cap) and P(kink) specific activities and apparent K_d_ values for SUVs. (**A**) Specific activities for X_PC_ = 0.0 (8 mM PI) and X_PC_ = 0.2 (8 mM PI/2 mM PC). (**B**) PI-PLC binding to DOPG/POPC SUVs as a function of X_PC_. Part (**A**) is modified, and part (**B**) is reprinted with permission from Cheng et al. Biophys. J. 2013, 104,185–195. Copyright 2013, Elsevier.

Based on these simulations, two proline residues in helix G are important for the clamshell motion: Pro245, termed Pro(cap) because it is at the N-terminal end of helix G, and Pro254, termed Pro(kink) because it is where the G-helix bends. The opening and closing of the loop linking helix F and the helix G loop with helix B is above the active site [27]. The mutation of Pro(cap) to Gly or Tyr reduced specific activities towards both PI and PI/PC SUVs (Figure 9A) and increased K_d_, the latter modestly (Figure 9B). Kinetic data for the soluble substrate cIP exhibited similar behavior. Pro(cap) is ~9 Å from the active site and adjacent to Tyr246; so, the substitution of that Pro could alter both the activity and the binding by altering the cation-π interaction. In contrast, the Pro(kink) mutants were not significantly affected in the absence or presence of PC. Simulations of both Pro mutants in solution showed that the clamshell motion was disrupted. The one anomaly in the simulations was that without Pro(kink), helix G was in an extended unkinked conformation that would allow access to the active site. This would yield specific activities and K_d_ values similar to those of the WT enzyme. 

Unlike many other members of the TIM barrel superfamily [28], *Bt*PI-PLC does not have a lid controlling access to its active site. Instead, the anticorrelated movement of the loops above the active site likely acts as a dynamic lid. PC molecules function as allosteric activators by anchoring the protein to the bilayer surface via formation of PC cation–*Bt*PI-PLC Tyr-π complexes and stabilizing an ‘open’ form of the protein where the PI can efficiently diffuse into the active site. The principle is the same for cIP hydrolysis. PC, whether in vesicles or micelles, stabilizes the enzyme in a state where the active site is accessible to the water-soluble substrate. Without the PC interface, the probability of a cIP molecule colliding with the protein when the clamshell is open and the active site is accessible is small. Locking *Bt*PI-PLC into an open conformation by binding PC leads to a large increase in k_cat_ for cIP. Consistent with this allosteric mechanism, soluble inhibitors based on the PI headgroup structure are poor inhibitors of the *Bt*PI-PLC catalyzed hydrolysis of cIP, but the presence of a PC surface (to which the protein binds) makes them much more potent [29]. In that assay system, neither the substrate nor the inhibitor partitions into the interface. The binding of *Bt*PI-PLC to PC and the formation of the two persistent cation-π complexes changes the dynamics of the protein to stabilize a conformation with an open active site.

The Dennis group has pioneered the concept that the membrane binding of several phospholipase A_2_ enzymes allosterically leads to an open conformation of the active site [30,31]. In fact, their recent computational and experimental study of lipoprotein-associated phospholipase A_2_ provides evidence that the membrane binding of the protein promotes a conformational change that opens access to the active site [32]. In contrast, in an aqueous environment, the positioning of a phospholipid in the active site is much less favorable. 

*Bt*PI-PLC is also allosterically affected by phospholipids. The *initial* binding of the protein does not require a large interfacial surface. Instead, two PC molecules form specific PC cation–Tyr-π complexes with the protein that then allow helix B and the β7-αG loop to insert into the bilayer. Rather than generating a significant conformational change in the protein, these complexes stabilize an open active site allowing processive catalysis of PI. The hydrolysis of soluble cIP is also enhanced because the active site is now open when the *Bt*PI-PLC is bound to a PC-containing surface.

## 5. Conclusions

These studies of *B. thuringiensis* PI-PLC have provided a number of insights into how PC cation–protein Tyr-π complexes can contribute to anchoring a peripheral membrane protein on PC-containing interfaces, which in turn can aid in the insertion of hydrophobic residues. Enzymatic activity is enhanced by these complexes stabilizing open access to the active site. The two cation-π sites prevent a clamshell motion that obstructs the active site when the protein is in solution. So far, there are only a few other peripheral membrane proteins where PC-cation–aromatic Tyr/Trp/Phe complexes have been identified and shown to be important for membrane binding. These include a number of phospholipases in addition to *Bt*PI-PLC: the cytosolic phospholipase A_2_, whose C_2_ domain forms a PC-cation–Tyr-π complex that is also stabilized by a Ca^+2^ interacting with the lipid phosphate group [33]; phospholipase A_2_ *Naja naja atra*, where ΔΔG data are available for replacing aromatic amino acids by Ala [34], and the PC specificity was shown to arise from cation-π complexes by simulations [26]; and a spider phospholipase D that uses Tyr cages around the bound PC cation [35]. The PC cation-π formation in the PLD appears conserved in many of the other members in the same clade, emphasizing these are distinct complexes that are useful for ensuring PC specificity. Simulations with PLD also showed that with a mixed PE/PC bilayer, no cation-π complexes were formed with PE. Neutrophil proteinase 3 [36], lung surfactant protein (SPA) [37], and equinatoxin, a soluble pore-forming toxin, [38] round out the group of interfacial enzymes with identified PC cation–aromatic amino acid π complexes. There is diversity in how many aromatic residues are involved in a PC cation–Tyr/Trp/Phe complex and in how many other residues contribute to stabilizing the complex. 

Two of these proteins, *Bt*PI-PLC and proteinase 3, both with verified PC cation–aromatic amino acid-π complexes, and the snake venom enzyme, *Naja naja* phospholipase A_2_, which has ΔΔG values for aromatic to Ala replacements binding to PC vesicles, were further examined with free energy perturbation simulations to see the variation in complex location in the bilayer, number, and choice of aromatic residues and the energy lost in the alanine mutants [39]. 

**Figure 10:**
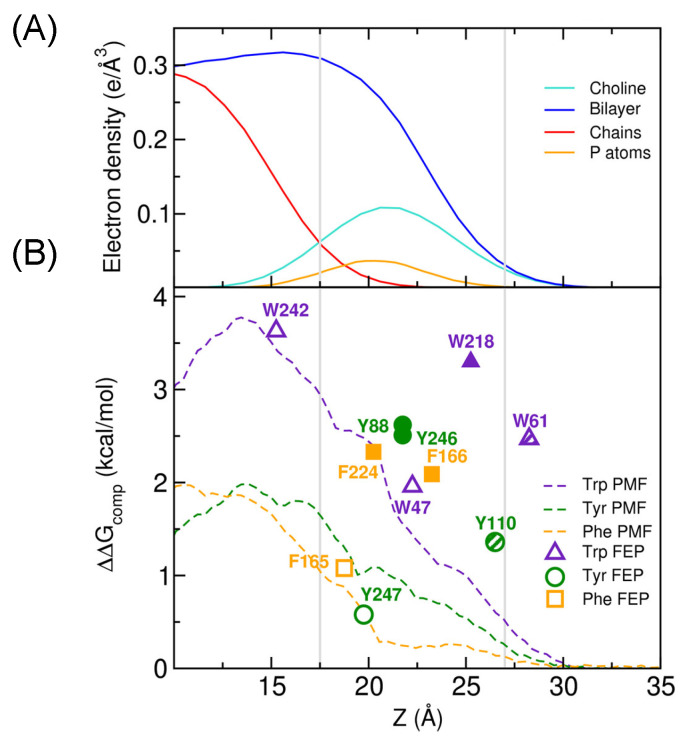
Computed ΔΔG_transfer_, which is ΔG_transfer_(Aro)–ΔG_transfer_(Ala), as a function of insertion depth, Z, the distance to the bilayer center. (**A**) Electron density profiles for different parts of phosphatidylcholine. (**B**) Free energy perturbation values for Trp (triangles), Tyr (circles), and Phe (squares). Filled symbols indicate amino acids with cation-π occupancy above 50%; hashed symbols indicate moderate cation-π occupancy (30–50%); empty symbols indicate occupancy <30%. PMF values are plotted as dashed lines. Reprinted with permission from Waheed et al. (2019) J. Phys. Chem. Lett. 10, 3972–3977. Copyright 2019, American Chemical Society.

Surprisingly, the interfacial aromatics mediating the cation-π interactions with choline-containing lipids can contribute as much to peripheral protein affinity for membranes as aromatics inserted below the phosphates. The π complexes show significantly higher free energy than the same amino acid in roughly the same location that is just partitioned in the membrane (compare F166 and F224, which have high occupancies for cation-π complexes, with F165 or Y88 and Y246 compared to Y247 in Figure 10). There is a cluster of Tyr or Phe cation-π complexes in the region of the phosphate and choline that have fairly similar ΔΔG values, but an energetically similar Trp cation-π complex (W61) can be significantly closer to the surface and still have a large ΔΔG values. Figure 10 emphasizes that aromatic amino acids are versatile in how and at what depth they can bind a PC headgroup and stabilize a peripheral membrane protein. Not only can the number and identity of aromatic amino acids vary, but the location of the complex can vary tremendously. The first region a protein will encounter on the way to the insertion of hydrophobic amino acids in a bilayer is that occupied by phospholipid headgroups. These studies strongly indicate that cation-π interactions of the protein with PC or other choline-containing lipids such as sphingomyelin are uniquely poised to help stabilize protein insertion at the upper region of the membrane interface. 

## 6. Appendix: Details on Analysis of ^31^P NMRDs and Examples

The field dependence of the dipolar relaxation of ^31^P by protons, R_D_, is described by the following equation:

(3) $$R_{D}=\left(\frac{R_{D}\left(0\right)}{2\tau_{D}}\right)(0.1\;J\left(\omega_{H}-\omega_{P}\right)+0.3\left(\omega_{P}\right)+0.6\;J\left(\omega_{H}+\omega_{P}\right)$$

where $J\left(\omega \right)=\frac{2\tau }{1+\omega_{2}\tau_{2}}$ J(ω) is the spectral density. The ω_P_ and ω_H_ are the gyromagnetic ratios for ^31^P and ^1^H, and τ_D_ and R_D_(0) are the correlation time and maximum relaxation rate for the dipolar interaction responsible for generating the NMRD. Each dipolar NMRD identified in a field cycling profile will be characterized by a τ_D_ and R_D_(0). Chemical shift anisotropy relaxation also contributes to the ^31^P relaxation, but only at high fields. The relaxation rate is described by Equation (4).

(4) $$R_{\mathrm{CSA}}=k_{\mathrm{CSA}}\frac{{\omega_{P}}^{2}{\tau_{\mathrm{CSA}}}^{2}}{1+{\omega_{P}}^{2}{\tau_{\mathrm{CSA}}}^{2}}≅k_{\mathrm{CSA}}\;{\omega_{P}}^{2}{\tau_{\mathrm{CSA}}}^{2}$$

If ω_P_^2^τ_CSA_^2^ << 1, then the expression can be simplified to a square law dependence of R_1_ on B_relax_. The k_CSA_ is related to the CSA interaction size and asymmetry. The ^31^P R_1_ data for dipalmitoylphosphatidylcholine in large unilamellar vesicles at 45 °C showed no deviation from a square law up to 21.3 T [40]; so, the approximation should be valid for the POPC/DOPMe SUVs used for the *Bt*PI-PLC studies. 

The total relaxation rate at each relaxation field, B_relax_, or Larmor angular frequency, ω_P_, is the sum of all the NMRD contributions. These small vesicles exhibit three dipolar and one CSA NMRD. The full R_1_ profile from 0.003 to 11.7 T is the sum of these four NMRDs (Equation (5)). For a more detailed look at how different NMRDs are related to phospholipid motions see [21].

R_1_ = R_D0_ + R_D1_ + R_D2_ + R_CSA_ (5)

Fitting the data is rarely performed for the entire field range. The region between 3 and 11.7 T is fitted as R_D2_(0) + R_CSA_; that sum is then is then subtracted at each field strength. What is left reflects R_D0_ + R_D1_. Since R_D0_ NMRD is where the spin label will have the largest effect, we usually fit the R_1_ data below 0.08 T as R_D0_ plus a constant (which would be R_D1_(0) + R_D2_(0)). After subtracting the data for the sample with the unlabeled protein from the data with the spin-labeled *Bt*PI-PLC in the low field region, the resultant ΔR_1(P-e)_ is fitted with a single spectral density term (Equation (6)) in order to extract the R_Do_ values for the ^31^P–electron dipolar interaction, τ_P-e_ and ΔR_P-e_(0), and estimate r_P-e_ (Equation (7)).

(6) $$\Delta R_{P-e}=\Delta R_{P-e}\left(0\right)/\left(1+{\omega_{P}}^{2}{\tau_{P-e}}^{2}\right)+c$$

(7) $${{{r_{P}}_{-}}_{e}}^{6}=([\mathrm{PLC}∙\mathrm{PL}]/[\mathrm{PL}{]}_{\mathrm{out}})\;\times \;\left(S^{2}\tau_{P-e}/\Delta R_{P-e}\left(0\right)\right){\left(\mu /4\pi \right)}^{2}\;{\left(h/2\pi \right)}^{2}\;{\gamma_{P}}^{2}{\gamma_{e}}^{2}$$

Equation (6) assumes that the ΔR_P-e_ represents the paramagnetic relaxation of a single bound POPC or DOPMe. Proximity of the spin-label to multiple PC binding sites (i.e., complexes with Tyr246 and Tyr88) can be done, but is more complex and the assumption of a single site is a good way to start. In Equation (7), μ_0_ is the magnetic permeability in a classical vacuum, h is Planck’s constant, and γ_e_ is the gyromagnetic ratio for an electron. The order parameter S^2^ is assumed to be 1 since faster motions that will only slightly change r_P-e_ on the µs timescale. Since *Bt*PI-PLC binds to the external leaflet of the vesicle, the total concentration of each type of phospholipid is multiplied by 0.67 to 0.75 to estimate [PL]_out_, the concentration of PC or PMe in the outer leaflet. For POPC/DOPMe (1:1) SUVs, the concentration of the phospholipids is sufficiently high compared to the apparent K_d_ so that all the enzyme will be partitioned on the SUVs. Therefore, [PLC∙PL] = [PLC]_o_ where [PLC]_o_ is the total concentration of enzyme added. While there are errors in τ_P-e_ and ΔR_P-e_(0), the ratio of the two is unlikely to be off by more than a factor of two. More importantly, the dependence of r_P-e_^6^ on τ_P-e_/ΔR_P-e_(0) means that the r_P-e_ extracted is fairly well defined.

**Figure 11:**
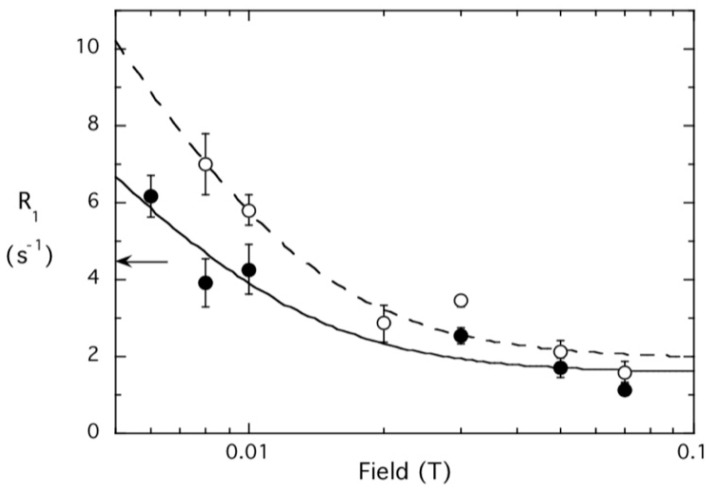
The R_D0_ region for the DOPMe ^31^P in SUVs of POPC/DOPMe (5 mM:5 mM) in the presence of 0.014 mM (filled circles) and 0.029 mM (open circles) spin-labeled *Bt*-PI-PLC H82C. The arrow indicates R_D0_(0) extrapolated to zero field for the SUVs with unlabeled *Bt*PI-PLC.

For many of the spin-labeled *Bt*PI-PLC cysteine variants, the low field dependence of R_1_ on B_relax_ was examined with a different protein concentration as a check on the extrapolated ΔR_P-e_(0). Figure 11 shows the R_D0_ NMRD for DOPMe with spin-labeled H82C at 0.014 and 0.029 mM. As a quick check, the difference in R_1_ between where the curves intersect the y-axis (it hasn’t reached R_D0_ yet) and the R_1_ at 0.1 T (where R_1_ is the sum of R_D1_(0) and R_D2_(0)) should differ about 2-fold). The τ_D0_ is similar for both samples and the ratio of R_D0_(0) for the two samples (1.7) is fairly close be proportional to the ratio of the protein concentration used (2.1).

**Figure 12:**
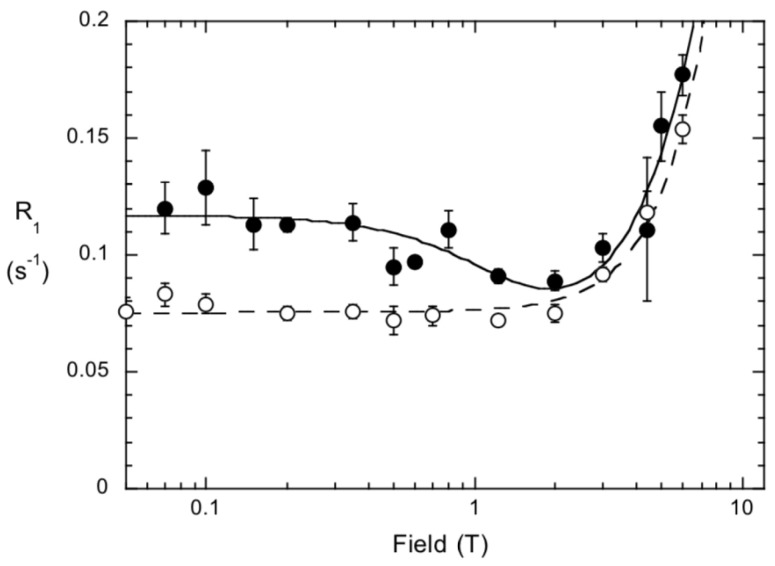
Dependence of ^31^P R_1_ on B_relax_ for diC_4_PC (5 mM) mixed with 0.0144 mM spin-labeled H82C (o) or D205C (•). The profile for diC_4_PC with labeled H82C is essentially the same as diC_4_PC without the protein.

^31^P field cycling with spin-labeled *Bt*PI-PLC has also been used to assess whether small molecules bind to the protein [41]. An example of this is shown in Figure 12. The protein was spin-labeled on D205C, near the proposed cation-π binding site, and on H82C, near the active site. The small molecule in solution has a field dependence of R_1_ that exhibits R_CSA_ at high B_relax_ and then a constant R_1_ that corresponds to the maximum R_D_(0) for fast motions. If it binds to a macromolecule, there should be a new NMRD with a correlation time closer to the rotational correlation time of the protein. If the R_1_ profile for the small molecule mixed with unlabeled protein in solution is subtracted, what is left represents the NMRDs for the effect of the spin-label unpaired electron attached to a protein Cys on the small molecule ^31^P. If there is no new NMRD, then the small molecule either does not bind or is too far away from the spin label. If the paramagnetic R_1_ enhancement increases as the concentration of diC_4_PC is increased, the site was initially not saturated. Figure 12 shows the effect of the two spin labels on the dC_4_PC ^31^P R_1_. There is virtually no effect of spin-labeled H82C on *Bt*PI-PLC. As expected this small water-soluble PC has no affinity for the *Bt*PI-PLC active site, nor does it bind to the Y88 cation-π site. However, there is a very small increase in R_1_ with spin-labeled D205C. The NMRD responsible for this increase has a 5–10 ns correlation time and an ΔR_P-e_(0) of 0.044 s^−1^. No lower field increase in R_1_ was observed, meaning any complexes that form do not persist more than ~10 ns. This indicates diC_4_PC can bind to the protein, presumably in the PC cation-Tyr246 π site, but the binding and occupancy are quite low.

## Author Information

^1^ Mary F. Roberts, Bryn Mawr College, 1969, A.B.; Stanford University, E.A. 1974, Ph.D.; University of Illinois, postdoctoral research with Dr. Robert Switzer (1974–1975); University of California San Diego, postdoctoral researcher with Dr. Edward Dennis (1975–1978)—almost 50 years ago (and she is still fascinated by phospholipases); M.I.T. Assistant/Associate Professor (1978–1986); Boston College, Associate Professor/Professor (1987–2017); Boston College, Professor of Chemistry Emerita (2017-present); ^2^ Anne Gershenson, Bryn Mawr College, 1990, A.B., University of Michigan, 1996, Ph.D. (physics); Caltech Postdoctoral Scholar (1997–2000); University of Illinois Urbana-Champaign Postdoctoral Research Associate (2000–2001); Assistant Professor, Brandeis University (2001–2009); Research Associate Professor, University of Massachusetts, Amherst, MA (2009–2021); present address: NIGMS/NIH Bethesda, MD 20892 USA; ^3^ Nathalie Reuter, Professor of Chemistry, University of Bergen; University Henri Poincaré-Nancy (France), 1999, PhD; Max Planck Society Fellow with Prof. Dr. Walter Thiel in 2000–2001 at MPI fuer Kohlenforschung (Muelheim-an-der-Ruhr).

## Notes

The results in this review article were collected while Anne Gershenson was associated with the University of Massachusetts Amherst. The opinions expressed in this article are the author’s own and do not reflect the view of the National Institutes of Health, the Department of Health and Human Services, or the United States government.
